# Relationship between ISO 9001:2015 and operational and business performance of manufacturing industries in a developing country (Indonesia)

**DOI:** 10.1016/j.heliyon.2020.e05537

**Published:** 2021-01-15

**Authors:** Rahmat Nurcahyo, Zulfadlillah, Muhammad Habiburrahman

**Affiliations:** Department of Industrial Engineering, Faculty of Engineering, Universitas Indonesia, Depok Campus, 16424, Indonesia

**Keywords:** Industrial engineering, Industry, Management, Industry management, Business management, Strategic management' ISO 9001, Operational performance, Business performance, Multiple linear regression analysis

## Abstract

Previous research has emphasized the need to further investigate the impact of ISO 9001 on company performance in the manufacturing sector of developing countries. Indonesia is one of those developing countries where the implementation of ISO 9001 is yet to be adequately researched. The Indonesian automotive manufacturing industry is still unable to compete with Malaysia and Thailand even though many companies have implemented ISO 9001. This study aimed to examine the relationship between ISO 9001 and operational (productivity, customer satisfaction, and product quality) and business (sales growth, profit rate, and market share) performance of Indonesian automotive component manufacturing industries. It also aimed to identify major obstacles in the effective implementation of ISO 9001. Multiple linear regression analyses about operational and business performance were employed for this purpose. The sample size comprised 50 automotive component manufacturing industries located in the Jakarta, Bogor, Tangerang, and Bekasi region of Indonesia. The study demonstrates that the implementation of the ISO 9001:2015 quality management system has a significant positive impact on the operational performance as well as the business performance. Additionally, the operational performance has a significant positive impact on the business performance. This study also reveals the major obstacles in the effective implementation of ISO 9001 in the manufacturing industry, which include a lack of qualified personnel, inadequate training, employee resistance, and lack of commitment among top-level management executives. It offers clear implications for managers who focus on elements that will enhance the effectiveness of ISO 9001 implementation by choosing the correct strategies, allocating sufficient resources, and improving their firm's performance. The novelty of this study lies in filling the existing research gap, which involves a detailed examination of the relationship between the implementation of ISO 9001 and the company's performance, particularly in manufacturing industries of developing countries.

## 1 Introduction

In the era of globalization, companies must focus on the quality of products and services provided to customers, in order to maintain their competitive advantage (Al-Najjar and Jawad, 2011). Quality is one of the key competitive strategies employed to improve company performance in the global market (Ismyrlis and Moschidis, 2015), which is an accurate representation of the fundamental condition of the company (Dobrin et al., 2015). Therefore, to enhance competitiveness, it is important for companies to foster a sense of innovation, and to focus on the quality of the products or services provided.

Quality management is defined as a systematic organization that ensures the implementation of an efficient process to achieve the company's goal (Taylor and Pearson, 1994). ISO 9001 is an international standard of quality management systems (Sari et al., 2017), which guarantees that the organization will provide products or services that meet the requirement of customers and relevant stakeholders (Yuri and Nurcahyo, 2013). The first ISO standard was published in 1987 by the International Organization for Standardization based in Geneva, Switzerland (Abraham et al., 2000). In 2015, the ISO 9001 was reviewed, and the latest version was introduced, namely ISO 9001:2015 (Chiarini, 2017) that emphasized the “process approach” and “risk-based thinking” in order to make the process stronger (Fonseca, 2015).

The effective implementation of ISO 9001 can provide a sustainable competitive advantage (Koc, 2007). ISO 9001 is useful for improving product quality (Mahadevappa and Kotreshwar, 2007) and services, as well as increasing quality awareness and control management (Brown et al., 1998). Camfield and Godoy (2004) stated that ISO 9001 is the industry standard for eliminating waste, improving productivity and efficiency, and increasing customer satisfaction (Cited in Almeida et al., 2018).

**Figure 1:**
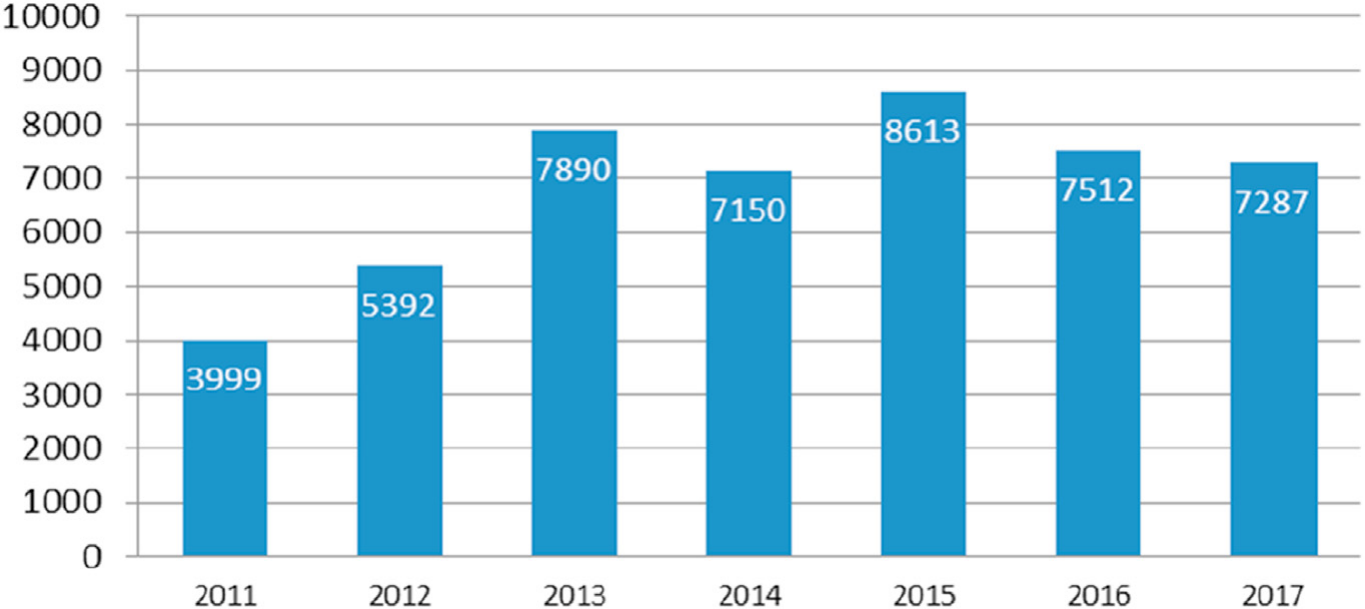
The number of companies that implement ISO 9001 in Indonesia.

The majority of ISO 9001 certified industries pertained to the manufacturing sector (Sumaedi and Yarmen, 2015). According to ISO survey data, in 2017, there were 7,287 industries in Indonesia that had implemented ISO 9001 (ISO, 2017). The yearly number of ISO 9001 certified Indonesian industries is shown in Figure 1.

The United Nations in the 45^th^ edition of the 2001 annual Statistical Yearbook divides the countries of the world into two groups: developed and developing countries (Mimba et al., 2007). Industrialization is seen as the most important driver of economic growth (Su and Yao, 2015), and the manufacturing sector in developing countries is responsible for sustainable economic growth (Haraguchi et al., 2016). The manufacturing sector is viewed as the leading edge of modernization and skilled job creation, as well as a fundamental source of various positive spillovers (Tybout, 2000). The manufacturing sector is one of the key catalysts for Indonesia's economic growth (Nurcahyo and Wibowo, 2015). Indonesia is categorized as a developing economy, where the manufacturing sector showed an average growth rate of 3.4% per year between 1991 and 2016 (UNIDO, 2018).

According to Kaplan (2011), performance can be defined as a set of financial and non-financial indicators that provide information on the proportion of goals and results that have been successfully achieved. Operational performance is related to the organization's internal operations, such as productivity, product quality, and customer satisfaction (Feng et al., 2007). Productivity is defined as a measure of the efficiency with which the input is converted into output (Reid and Sanders, 2011). Productivity measures how well resources can be utilized and is calculated as the ratio of output (goods and services) to input (e.g. labor and material). The productivity of a company is directly proportional to its efficiency. Quality is defined as fitness for use, conformance to requirements, and zero defects (Taylor and Pearson, 1994), while customer satisfaction is generally interpreted as a reaction of customers to the condition of fulfillment (Kim et al., 2004). Business performance is defined as the operational ability to fulfill the wishes of the company's main shareholders and is a dependable indicator of the performance of an organization (Zulkiffli and Perera, 2011). Business performance can be measured by financial and marketing performance parameters such as sales growth, profit level, and market share (Feng et al., 2007).

Manufacturing performance is integral to the success of companies where superior performance leads to an increase in competitiveness (Amrina and Yusof, 2011). In the manufacturing sector, it is important for companies to identify and evaluate the parameters that improve their performance, especially those related to operational performance (Tan and Wong, 2015). The main objective of a manufacturing organization should be to improve its operational performance (Ali et al., 2020). The most commonly used measurements of operational performance are quality, time and delivery, cost, flexibility, customer satisfaction (El Mola and Parsaei, 2010), and productivity (Feng et al., 2007). An analysis of the customer satisfaction concept concerning quality issues has been radically evolved (Reeves and Bednar, 1994) from conformance to-specifications towards a more consumer-based definition (Muffatto and Panizzolo, 1995). The similarities between conformance to-specifications and consumer-based are a) both are perspectives of quality, and b) both can be used to measure the quality of a product and service (Reeves and Bednar, 1994). However, there are some differences between them. While conformance to-specifications is the early perspective on quality that begins in the manufacturing industry, consumer-based perspective emerged recently to better explain the quality in the service industry (Reeves and Bednar, 1994). This study uses the conformance to-specifications perspective since it was developed in the context of manufacturing firms (Muffatto and Panizzolo, 1995). Feng, Terziovski & Samson (2007) stated that, in manufacturing industry, customer satisfaction variable belongs to operational performance. Therefore in this study, the customer satisfaction is part of operational performance. Company performance can be measured on the basis of business performance, which is related to sales growth, profit level, and market share (Feng et al., 2007).

Global competition encourages manufacturing industries to enhance their competitiveness on a multidimensional scale (Desai and Prajapati, 2017). To increase its competitive advantage, the manufacturing industry must rely on proper quality procedures (Saleh et al., 2018) and implement a robust Quality Management System (Priede, 2012). The importance of the ISO 9001 based Quality Management System is highlighted by the fact that it helps the manufacturing industries to gain a significant competitive advantage over others (Magd and Curry, 2003; Kaziliunas, 2010).

The manufacturing industry is driving Indonesia's economic growth with a contribution to GDP reaching 22% in 2016 (Bank Mandiri, 2018). The Indonesian automotive industry is a manufacturing sub-industry that has significant growth. in 2015 the value of investment realization was 1757 Million USD (The Ministry of Industry, 2018). However, this automotive investment is only in the third position in the South East Asian region because it is unable to compete with Malaysia and Thailand (Nurcahyo and Wibowo, 2015).

Multiple studies have been conducted on the impact of ISO 9001 on company performance, but due to the varying nature of the aspects under consideration, the results were quite different from each other (Martin, 2017). Some of these studies concluded that ISO 9001 had a positive impact, while others opined that there was no discernible impact on company performance (Kumar et al., 2018). It was observed that ISO 9001 had a positive impact on the operational and business performance of the Spanish furniture industry (Marin and Ruiz-Olalla, 2011), the Guyanese manufacturing industry (Wilcock and Boys, 2017), and the Malaysian manufacturing industry (Tan and Sia, 2001). ISO 9001 had also improved the business performance of the Algerian (Yahia-Berrouiguet et al., 2015) and Tanzanian (Mangula, 2013) manufacturing industries. The quality management system directly impacted the operational performance, but did not affect the business performance of the manufacturing industries from Greece (Kafetzopoulos et al., 2015), Australia and New Zealand (Feng et al., 2007). ISO 9001 had a partial impact on the business performance (return on net assets), but did not affect the profit and revenue streams of Kenyan industries (Ochieng et al., 2015). ISO 9001 impacted the operational performance of Pakistani manufacturing industries (Mahmood and Hasan, 2012). Singh, Bhardwaj, and Sachdeva (2007) stated that ISO 9001 did not have a significant impact on some aspects of operational performance in the Indian manufacturing industry. Wu and Wu (2019) stated that ISO 9001 had a positive effect on product innovation in an emerging market.

The researchers stated the need to conduct further studies on the impact of ISO 9001 on company performance in the manufacturing sector of other developing countries (Ahmed, 2017; Singh et al., 2018; Kumar et al., 2018). Indonesia is included in that list (Amar and Zain, 2002); however, only a few papers have been published regarding the implementation of ISO 9001 in Indonesia. Alfredo and Nurcahyo (2018) researched the impact of the integrated management system (ISO 9001, ISO 14001 and OHSAS 18001) on the operational performance of manufacturing industries, taking into account parameters such as production volume, production efficiency and waste reduction. Among other researchers, Wahyudi et al. (2012) developed a research framework about the impact of quality management practices on business performance by using two mediating factors (organizational culture and competitive attributiveness of product quality). Therefore, the importance of research regarding the relationship of ISO 9001 with operational and business performance cannot be overstated. Keeping this in mind, we have attempted to examine the relationship of ISO 9001 with the operational and business performance of manufacturing companies in a developing country (i.e., Indonesia).

## 2 Material and methods

In the current business environment, there is increasing pressure on companies from consumers and competitors to focus on innovative products and to improve the quality of goods and services. As a result, most companies in both developed and developing countries have adopted several forms of ISO certification (Ochieng et al., 2015). ISO 9001 is an international quality management system standard that supports companies in their efforts to improve management practices. By combining elements such as continuous improvement, process management, leadership, and customer satisfaction focus into the management system, the adoption of this standard will encourage enhanced efficiency and profits for the company. The applicability of this standard is not restricted to a particular industry or country (Wilcock and Boys, 2017). By providing relevant principles to guide the company's processes, this standard enables companies from developed as well as developing countries to compete in the international market.

Therefore, future research endeavors on this matter can concentrate entirely on developing countries because these regions are poised to become global manufacturing centers (Kumar et al., 2018). Developed countries are increasingly abandoning manufacturing industries in favor of service-based industries. This behavioral shift can be attributed to the fact that manufacturing industries are responsible for air pollution. Additionally, limited workforce availability in developed countries is another deterrent, as compared to the labor-rich developing countries. Manufacturing industries tend to be labor-intensive, while service-based industries are more profitable and require less manpower. The high level of education in developed countries is more suitable for the service industry, as compared to the manufacturing industry.

**Figure 2:**
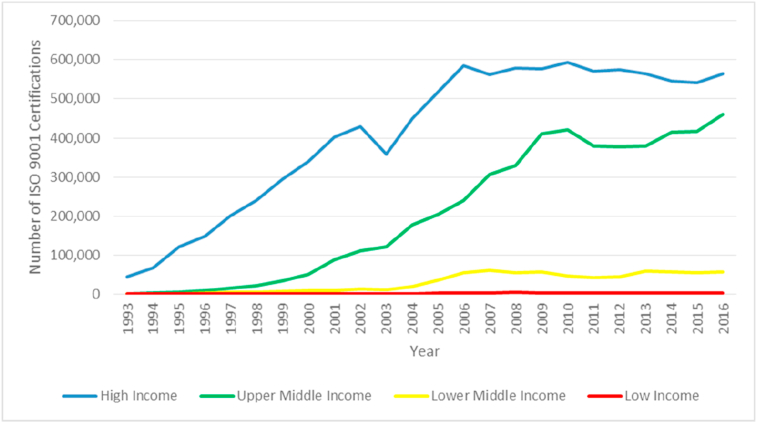
Number of ISO 9001 certifications from 1993 to 2016 (by income groups).

United Nations (2018) classifies on the basis of Gross National Income into four categories: low-income, lower-middle-income, upper-middle-income, and high-income. Low-income countries include Afghanistan, Ethiopia and Somalia. Lower-middle-income countries include Indonesia, India, Nigeria and Bolivia. Upper-middle-income countries include Malaysia, Turkey, Mexico, Brazil, and Thailand. Countries that are included in the high-income category include Japan, UK, South Korea, Germany and the USA. The number of ISO 9001 certifications in high-income, middle-income, and low-income groups in the period from 1993 to 2011 is shown in Figure 2.

**Figure 3:**
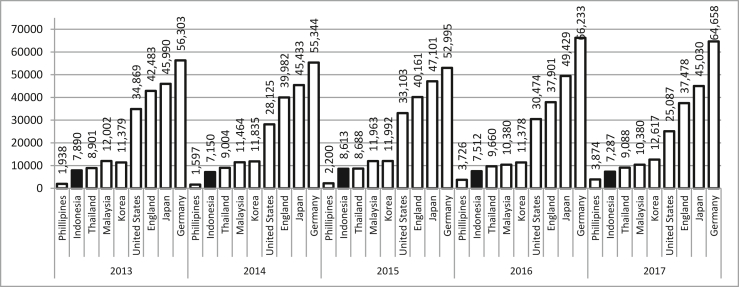
Comparison between the number of ISO 9001 certifications in ASEAN-4 and developed countries.

The number of ISO 9001 certifications in ASEAN-4 (Philippines, Indonesia, Malaysia, and Thailand) is compared with developed countries such as South Korea, America, England, and Japan in the last five years from 2013 to 2017 in Figure 3 (ISO, 2017). It may be observed from Figure 3 that Indonesia is always ranked third based on the number of ISO 9001 certifications. In 2017, there were 10,380, 9,088, 7,287 and 3,874 ISO 9001 certified companies in Malaysia, Thailand, Indonesia and Philippines, respectively. As for the developed countries, in 2017 there were 12,617, 25,087, 37,478, 45,030 and 64,658 ISO 9001 certified companies in South Korea, the United States, the UK, Japan and Germany, respectively.

The implementation of ISO 9001 in developing and developed countries with respect to operational performance (product quality, productivity and customer satisfaction) and business performance (sales growth, profit rates and market share) was studied. In a developing country, implementing ISO 9001 can improve firm performance parameters such as product quality, customer satisfaction, productivity, profit and market share (Tan and Sia, 2001; Singh et al., 2007; Mahmood and Hasan, 2012; Mangula, 2013; Ochieng et al., 2015; Yahia-Berrouiguet et al., 2015). Marin and Ruiz-Olalla (2011) stated that, in a developed country, ISO 9001 can improve quality of the product and enhance customer satisfaction. Heras, Dick, and Casadesus (2002) stated that ISO 9001 has a positive impact on sales growth and profit. Corbett et al. (2005) stated that ISO 9001 has a positive impact on increasing productivity and sales growth.

While researching the relationship between quality management system and firm performance, it is imperative to determine the dependent and independent variables. Operational performance and business performance are defined as dependent variables, while quality management principles are classified under independent variables. The operational performance, business performance and the ISO 9001 variables are shown in Table 1.

**Table 1:** Operational performance, Business performance and ISO 9001 variables.

Operational Performance Variables	References
Productivity	Magd and Curry (2003), Feng et al. (2007), Avella and Vazquez-Bustelo (2010), El Mola and Parsaei (2010), Kim et al. (2011), Bolboli and Reiche (2013), Kafetzopoulos et al. (2015), Yahia-Berrouiguet et al. (2015), Magd, 2006
Customer satisfaction	Brown et al. (1998), Magd and Curry (2003), Feng et al. (2007), Singh et al. (2007), Han et al. (2009), El Mola and Parsaei (2010), Bolboli and Reiche (2013), Yahia-Berrouiguet et al. (2015), Magd, 2006
Product quality	Brown et al. (1998), Lee et al. (2001), Magd and Curry (2003), Liao et al. (2004), Lakhal et al. (2006), Feng et al. (2007), Singh et al. (2007), Han et al. (2009), El Mola and Parsaei (2010), Mangula (2013), Yahia-Berrouiguet et al. (2015), Magd, 2006.
Business Performance Variables	References
Sales growth	Feng et al. (2007), Han et al. (2009), Avella and Vazquez-Bustelo (2010), Kafetzopoulos et al. (2015), Yahia-Berrouiguet et al. (2015), Magd, 2006.
Profit rate	Singh and Smith (2006), Feng et al. (2007), Han et al. (2009), Kafetzopoulos et al. (2015), Yahia-Berrouiguet et al. (2015), Magd, 2006.
Market share	Brown et al. (1998), Singh and Smith (2006), Feng et al. (2007), Han et al. (2009), Avella and Vazquez-Bustelo (2010), Kafetzopoulos et al. (2015), Yahia-Berrouiguet et al. (2015), Magd, 2006.
ISO 9001 Variables	References
Customer requirement	Lakhal et al. (2006), Singh and Smith (2006), Sadikoglu and Zehir (2010), Kim et al. (2011), Psomas et al., 2013, Kafetzopoulos and Gotzamani (2014), Kafetzopoulos et al. (2015).
Defect prevention	Avella and Vazquez-Bustelo (2010), Marin and Ruiz Olalla (2011), Psomas et al. (2011; 2013), Kafetzopoulos and Gotzamani (2014), Kafetzopoulos et al. (2015).
Continuous improvement	Singh and Smith (2006), Su et al. (2008), Sadikoglu and Zehir (2010), Avella and Vazquez-Bustelo (2010), Psomas et al. (2011; 2013), Kim et al. (2011), Kafetzopoulos and Gotzamani (2014), Kafetzopoulos et al. (2015).
Organizational leadership	Amar and Zain (2002), Feng et al. (2007), Sadikoglu and Zehir (2010), Kim et al. (2011).
Supplier quality management	Lakhal et al. (2006), Singh and Smith (2006), Sadikoglu and Zehir (2010), Wilcock and Boys (2017).

Based on the literature review above, the research hypothesis can be constructed as follows:

H1 ISO 9001 has a positive impact on company operational performance

H2 ISO 9001 has a positive impact on company business performance

H3 Operational performance has a positive impact on business performance

**Figure 4:**
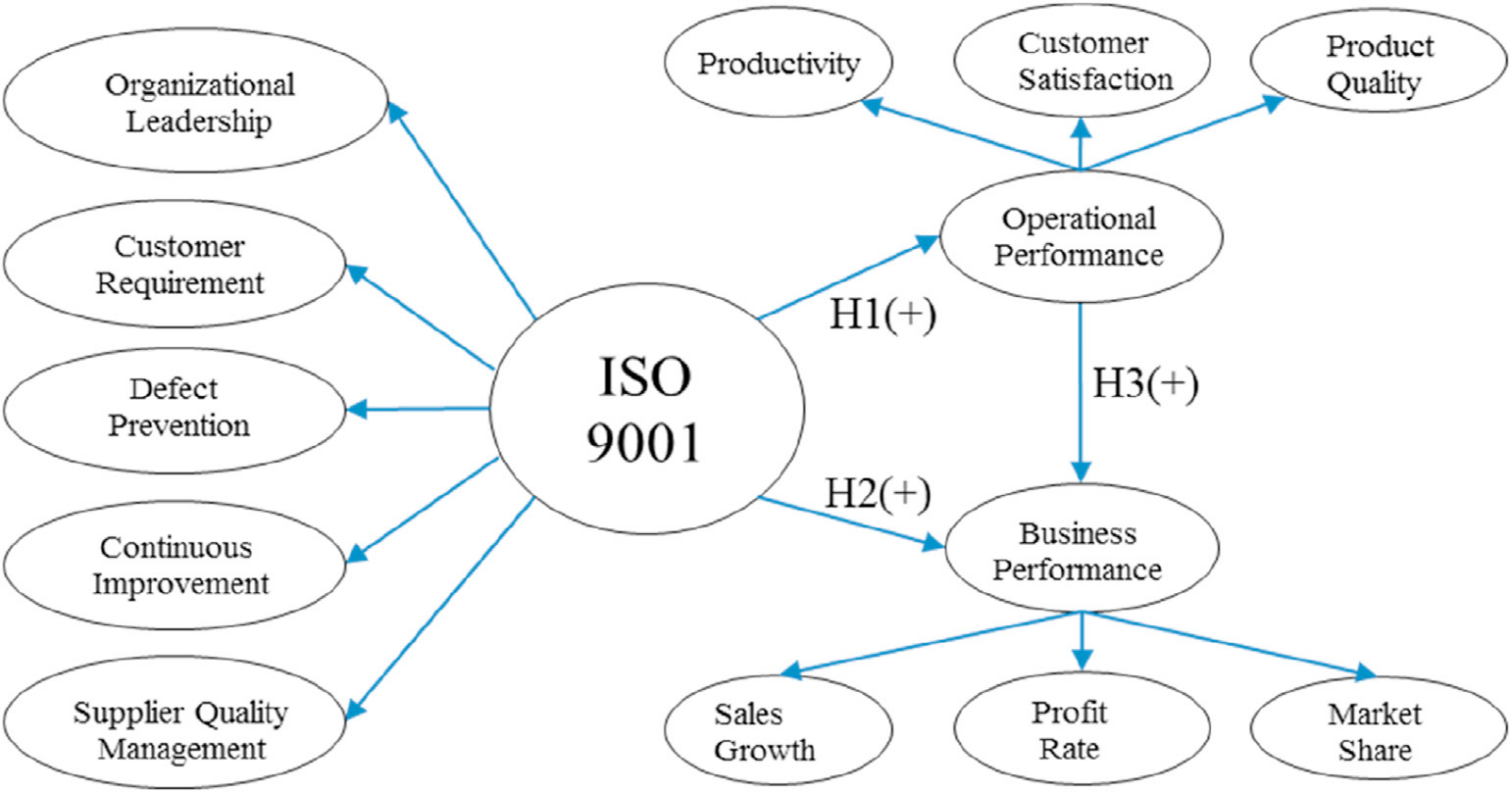
Research conceptual model.

After constructing the research's conceptual model (Figure 4), the next step is creating the questionnaire for data collection. There is no agreement on the number of scale points to be used; most studies use four to seven points. Only the six-point scales follow normal distributions statistics (Leung, 2011). Each question in the questionnaire uses a six-point Likert scale (1 = strongly disagree, 2 = disagree, 3 = somewhat disagree, 4 = somewhat agree, 5 = agree, and 6 = strongly agree). The questionnaire is divided into sections. Section 1 involves general data/demographic information, while section 2 deals with ISO 9001 principles and company performance assessment(s). The operational and business performance assessments use a six-point Likert scale (1 = very low from the target and 6 = exceeded the target by a substantial margin). The questionnaire was subsequently distributed to 30 respondents as a pilot project (Table 2), to check the validity and reliability of the questionnaire, post which it was distributed to 50 companies (Table 3). The data was processed via multiple linear regression analysis, using IBM SPSS Statistics 25 software.

**Table 2:** Descriptive statistics for the validity and reliability test (pilot test).

		n	%
Company age	<5 years	1	3.3%
	5–10 years	1	3.3%
	>10 years	28	93.3%
Number of employees	<100 Employees	2	6.7%
	100 - 500 Employees	11	36.7%
	>500 Employees	17	56.7%
Time period in applying ISO 9001	<3 years	3	10.0%
	3–6 years	2	6.7%
	>6 years	25	83.3%
Respondent age	<30 years	4	13.3%
	30–40 years	17	56.7%
	>40 years	9	30.0%
Respondent position in the company	Assistant Manager	14	46.7%
	Manager	14	46.7%
	General Manager	2	6.7%
Respondent working experience	<5 years	3	10.0%
	5–10 years	7	23.3%
	>10 years	20	66.7%

**Table 3:** Descriptive statistics for regression analysis (50 companies).

		n	%
Company age	<5 years	1	2%
	5–10 years	3	6%
	>10 years	46	92%
Number of employees	<100 Employees	2	4%
	100 - 500 Employees	17	34%
	>500 Employees	31	62%
Time period in applying ISO 9001	<3 years	5	10%
	3–6 years	4	8%
	>6 years	41	82%
Respondent age	<30 years	5	10%
	30–40 years	25	50%
	>40 years	20	40%
Respondent position in the company	Assistant Manager	14	28%
	Manager	30	60%
	General Manager	6	12%
Respondent working experience	<5 years	5	10%
	5–10 years	8	16%
	>10 years	37	74%

For deeper understanding of the statistical result, this study also conducted a survey regarding the barriers in the effective implementation of the ISO 9001 quality management system. Previous studies were used to develop the questionnaire. Thirty manufacturing companies were selected to be the respondents which belonged to the managerial level.

## 3 Results

### 3.1 Result of validity testing

Validity is the degree to which a scale or set of measures accurately represents the concept of interest (Hair et al., 2010). The validity test in this research employed the Bivariate Pearson Correlation technique with the IBM SPSS Statistics 25 software. If the Pearson Correlation value > r Table 0.361 (n = 30), then the question item is valid. The validity test invalidated question number 5 of the organizational leadership variable (Pearson correlation = 0.243). This question item is removed from the questionnaire.

### 3.2 Result of reliability testing

Reliability is an assessment of the degree of consistency between multiple measurements of variables. Reliability tests with Cronbach's alpha is the most widely used tool. The generally consensus for the lower limit of Cronbach's alpha is 0.6 in an exploratory study (Hair et al., 2010). Cronbach's alpha value for the entire questionnaire is shown in Table 4, and Cronbach's alpha value for each variable is shown in Table 5. According to Table 4, Cronbach's alpha value is 0.948. It concludes that the questionnaire is highly reliable.

**Table 4:** Reliability test results.

Reliability Statistics	
Cronbach's alpha	N of Items
0.948	26

**Table 5:** Reliability test result of each variable.

Variables	No. of Items	Cronbach's alpha	Reliability
Organizational Leadership	6	0.672	Reliable
Customer Requirement	5	0.873	Reliable
Defect Prevention	5	0.919	Reliable
Continuous Improvement	5	0.926	Reliable
Supplier Quality Management	5	0.902	Reliable

Table 5 shows the Cronbach's alpha of each variable. The continuous improvement variable has the highest Cronbach's alpha value of 0.926, and the organizational leadership variable has the lowest Cronbach's alpha value of 0.672. Thus, it may be concluded that the continuous improvement variable has maximum reliability, and the organizational leadership variable has minimum reliability.

### 3.3 Result of hypotheses testing

#### 3.3.1 Result of the regression of ISO 9001 on operational performance (H1)

According to Montgomery (2013), if F value < F table, then H_0_ is accepted, implying that the independent variable does not significantly affect the dependent variables. Moreover, if F value > F table, then H_0_ is rejected, implying that at least one of the independent variables affect the dependent variable significantly. To answer the hypotheses H1 and H2, we must determine the F table by the formulation F (k; n-k), where k = number of independent variables, and n = number of samples. Thus, we obtain the F table of F (5; 50-5) = F (5; 45) = 2.42.

Based on the result of the ISO 9001 regression test on operational performance as shown in Table 6, the F value = 4.739 > F table = 2.42 and the significance value 0.002 < 0.05. Thus, it can be concluded that all ISO 9001 variables simultaneously affect the operational performance. The determination coefficient in linear regression is defined as the ability of all independent variables to explain the variance of the dependent variable. Based on Table 7, the R-square value is 0.35 (35%). It means that all ISO 9001 variables simultaneously influence the operational performance variable by 35%. According to Hair et al. (2010), if the number of samples n = 50, and the number of independent variables is 5, the minimum R square value that can be obtained is statistically significant at 23%. Thus, the determination coefficient of 35% shows that the regression model in this study is acceptable.

**Table 6:** Regression of ISO 9001 on operational performance.

ANOVA^a^						
Model		Sum of Squares	Df	Mean Square	F	Sig.
1	Regression	82.862	5	16.572	4.739	.002^b^
	Residual	153.858	44	3.497		
	Total	236.720	49			

a Dependent Variable: Operational performance.

b Predictors: (Constant), Supplier Quality Management, Defect Prevention, Organizational Leadership, Continuous Improvement, Customer Requirement.

**Table 7:** Determination of the coefficient of regression of ISO 9001 on operational performance.

Model Summary^b^				
Model	R	R Square	Adjusted R Square	Std. Error of the Estimate
1	.592^a^	.350	.276	1.86997

a Predictors: (Constant), Supplier Quality Management, Defect Prevention, Organizational Leadership, Continuous Improvement, Customer Requirement.

b Dependent Variable: Operational Performance.

#### 3.3.2 Result of the regression of ISO 9001 on business performance (H2)

Based on the results of the ISO 9001 regression test on business performance in Table 8, the F value = 4.063 > F table = 2.42 and the significance value 0.004 < 0.05. Thus, it can be concluded that all ISO 9001 variables simultaneously affect the business performance. Based on Table 9, the R-value is equal to 0.562, so that the R-square value is 0.316 (31.6%). It means that all ISO 9001 variables simultaneously influence the business performance by 31.6%. Thus, the coefficient of determination of 31.6% shows that the regression model in this study is acceptable.

**Table 8:** Regression of ISO 9001 on business performance.

ANOVA^a^						
Model		Sum of Squares	Df	Mean Square	F	Sig.
1	Regression	121.732	5	24.346	4.063	.004^b^
	Residual	263.648	44	5.992		
	Total	385.380	49			

a Dependent Variable: Business Performance.

b Predictors: (Constant), Supplier Quality Management, Defect Prevention, Organizational Leadership, Continuous Improvement, Customer Requirement.

**Table 9:** Determination coefficient of regression of ISO 9001 on business performance.

Model Summary^b^				
Model	R	R Square	Adjusted R Square	Std. Error of the Estimate
1	.562^a^	.316	.238	2.44786

a Predictors: (Constant), Supplier Quality Management, Defect Prevention, Organizational Leadership, Continuous Improvement, Customer Requirement.

b Dependent Variable: Business Performance.

#### 3.3.3 Result of the regression of operational performance on business performance (H3)

To answer the hypotheses H3, we determined the F table by the formulation F (k; n-k), where k = number of independent variables, n = number of samples. Therefore, we obtain the F table of F (3; 50-3) = F (3; 47) = 2.8. Based on the regression test result of operational performance on business performance as shown in Table 10, the F value = 13.443 > F table = 2.8, and the significance value 0.000 < 0.05. Thus, it can be concluded that operational performance significantly affects business performance. Based on the output of the calculation results in Table 11, the R-square value is 0.467 (46.7%). This implies that operational performance affects business performance by 47.2%.

**Table 10:** Regression of operational performance on business performance.

ANOVA^a^						
Model		Sum of Squares	Df	Mean Square	F	Sig.
1	Regression	135.066	3	45.022	13.443	.000^b^
	Residual	154.054	46	3.349		
	Total	289.120	49			

a Dependent Variable: Business Performance.

b Predictors: (Constant), Customer Satisfaction, Productivity, Product Quality.

**Table 11:** Determination of coefficient of operational performance and business performance.

Model Summary				
Model	R	R Square	Adjusted R Square	Std. Error of the Estimate
1	.683^a^	.467	.432	1.83003

a Predictors: (Constant), Customer Satisfaction, Productivity, Product Quality.

#### 3.3.4 Correlation matrix

Based on the results of the ISO 9001 correlation test on operational performance and business performance shown in Table 12 of the correlation matrix, Pearson correlation values obtained from all ISO 9001 variables on operational performance and business performance are positive. Thus, it can be stated that all ISO 9001 variables have a positive impact on operational performance and business performance.

**Table 12:** Correlation matrix of ISO 9001 variables with Operational Performance and Business Performance.

Correlations			
		Operational Performance	Business Performance
Organizational Leadership	Pearson Correlation	0.47	0.326
	Sig. (2-tailed)	0.001	0.021
	N	50	50
Customer Requirement	Pearson Correlation	0.461	0.387
	Sig. (2-tailed)	0.001	0.006
	N	50	50
Defect Prevention	Pearson Correlation	0.414	0.379
	Sig. (2-tailed)	0.003	0.007
	N	50	50
Continuous Improvement	Pearson Correlation	0.517	0.285
	Sig. (2-tailed)	0	0.045
	N	50	50
Supplier Quality Management	Pearson Correlation	0.525	0.533
	Sig. (2-tailed)	0	0
	N	50	50

Based on the results of the operational performance correlation test on business performance as shown in Table 13 of the correlation matrix, obtained Pearson correlation values of operational performance variables on business performance are positive. Thus, it can be stated that operational performance has a positive relationship with business performance.

**Table 13:** Correlation matrix of operational performance variables with business performance.

Correlations		
		Business Performance
Productivity	Pearson Correlation	0.646
	Sig. (2-tailed)	0
	N	50
Product Quality	Pearson Correlation	0.551
	Sig. (2-tailed)	0
	N	50
Customer Satisfaction	Pearson Correlation	0.501
	Sig. (2-tailed)	0
	N	50

## 4 Discussion

From the results of regression analysis, it can be concluded that all ISO 9001 variables simultaneously affect the operational performance and business performance. In addition, operational performance significantly affects and has a positive relationship with business performance. It also can be stated that all ISO 9001 variables have a positive impact on operational performance and business performance.

The results of this study support previous several studies in developing countries such as Algeria and Malaysia that the implementation of ISO 9001 positively affects product quality, customer satisfaction, productivity and market share. The type of industry that is the object of research in Algeria and Malaysia is different from this research. In Algeria, it is carried out in the cement industry while in Malaysia, it is carried out in the electronic industry, however, Algeria and Malaysia have similarities with Indonesia as developing countries (Yahia-Berrouiguet et al. (2015) dan Tan and Sia (2001)).

Companies in developing countries have been reported to experience multiple difficulties in the implementation of a quality management system. Research in Indonesia (Amar and Zain, 2002) has shown that a lack of commitment at the top-level management, and a general lack of personnel competency are the main obstacles to the proper implementation of ISO 9001. In developing countries, the top management executives may not fully comprehend the importance of meeting customer requirements and developing good relationships with suppliers in order to secure the supply of raw material. In addition, several companies in developing countries deliver poor quality raw materials that are uncontrolled, and also suffer from inadequate maintenance of equipment (Wilcock and Boys, 2017). According to Al-Najjar and Jawad (2011), the barriers in the implementation of the ISO 9001 quality management system in developing countries include lack of commitment at the top management, employee resistance (implying difficulty in changing the mindset of employees about the importance of quality), lack of qualified personnel (competence), inadequate training, lack of financial resources, unrealistic quality management system requirements, difficulties in carrying out an internal audit, and a marked absence of consulting institutions.

On the basis of the aforementioned obstacles, this study also conducted a survey regarding the barriers in the effective implementation of the ISO 9001 quality management system. The questionnaire was distributed to 30 manufacturing companies, who had already implemented the ISO 9001 quality management system; the respondents belonged to the managerial level. Sixteen companies (53%) responded to the survey, the results of which are is expected help in determining the main obstacles faced by the Indonesian manufacturing industry. The survey used a questionnaire technique (YES/NO questions) and consisted of the list of obstacles.

**Figure 5:**
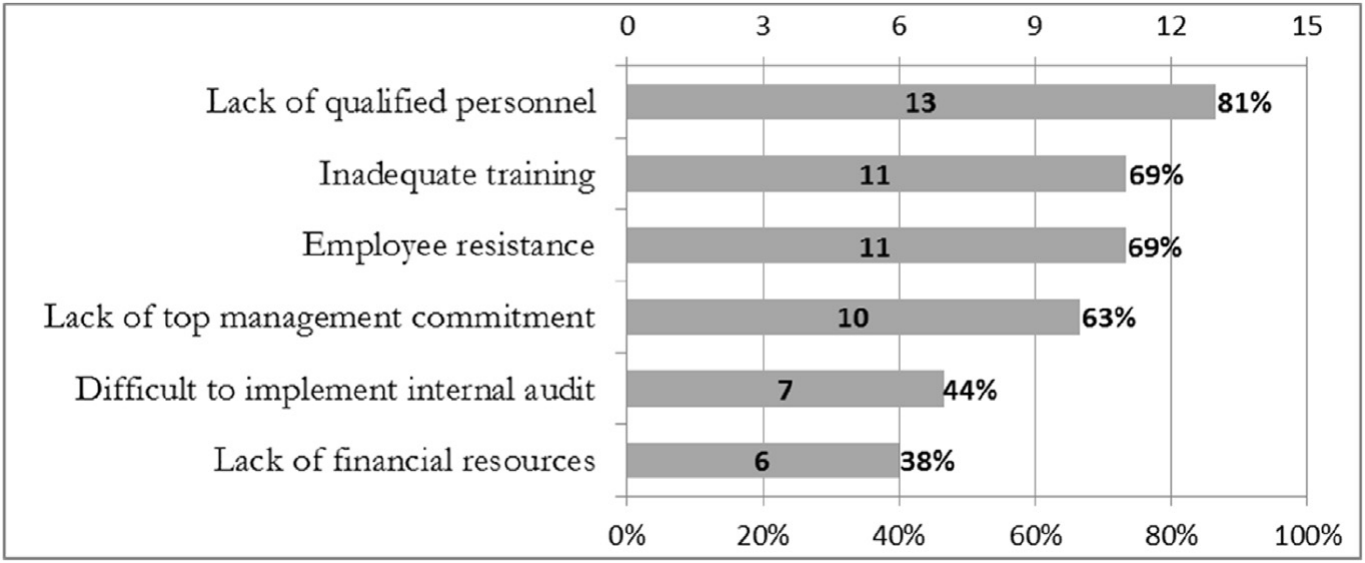
Obstacles in the implementation of ISO 9001.

For the implementation of the ISO 9001 quality management system in the Indonesian manufacturing industry, the lack of qualified personnel proved to be the predominant obstacle, with 81% (or 13 respondents) answering "Yes". The second biggest obstacle was inadequate training, with 69% (or 11 companies) answering "Yes". Other factors included employee resistance (69%, or 11 companies answering "Yes"), lack of commitment at the top-level management (63%, or 10 companies answering "Yes"), difficulties in carrying out internal audit (44%, or 7 companies answering "Yes"), and lack of financial resources (38%, or 6 companies answered "Yes"). These results can be seen in Figure 5.

Lack of qualified personnel is related to the lack of information, and proper understanding of ISO 9001 quality management system requirements. Inadequate training is related to the lack of a robust ISO 9001 quality management system training program. Employee resistance is an obstacle caused by difficulties in changing the mindset of employees about the importance of quality. The lack of commitment at the top-level management executives stems from their lackadaisical attitude towards quality. If they are highly dedicated to quality programs, it will foster an atmosphere of continuous improvement and encourage the participation of all employees in quality programs. Difficulties in implementing internal audits are related to a lack of understanding of the quality management system, the level of education of employees, and lack of training. Lack of financial resources is related to the costs needed for training programs, provision of resources related to quality, consultant financing, auditor financing, and funding for certification (Al-Najjar and Jawad, 2011).

## 5 Conclusions

The company's operational performance and business performance are of paramount importance in increasing the global competitive advantage of the manufacturing industry. One of the factors that can affect operational and business performance is the implementation of the ISO 9001 quality management system. This study concludes that all ISO 9001 variables have a positive impact to the operational performance and business performance. The ISO 9001 variables are organizational leadership, customer requirement, defect prevention, continuous improvement and supplier quality management. Furthermore, operational performance has a positive impact to business performance. This research supports previous studies regarding the impact of ISO 9001 in manufacturing industries, especially in developing countries. This research also revealed several obstacles faced by companies during the implementation of ISO 9001, which include lack of qualified personnel, inadequate training, employee resistance and lack of commitment from the top-level management. Thus, this research can be used as a reference by companies to implement the ISO 9001 quality management system effectively, which will enhance the operational and business performance of the manufacturing industry.

The novelty of this study lies in filling the existing research gap, which involves a detailed examination of the relationship between the implementation of ISO 9001 and the company's performance, particularly in manufacturing industries of developing countries. This paper is limited to the manufacturing industry and it is possible that other industry sectors would show significant differences. Current research is based on quantitative data processing using a statistical approach. It will be better if it is equipped with qualitative research to enrich quantitative results. Qualitative research includes FGDs and deep interviews. Also, other perspectives of operational and business performance that applied in the service industry could lead to different results. The sample of this study were 50 automotive manufacturing companies. Future studies can increase the number of samples because more research samples will provide a better confidence level in the research results. Future research is also strongly suggested examining the relationship between ISO 9001: 2015 and the operational and business performance of manufacturing industries in other developing countries. Moreover, using a consumer-based approach to examine the relationship between ISO 9001: 2015 and company performance of service industries would contribute to the existing knowledge.

## Declarations

### Author contribution statement

Rahmat Nurcahyo: Conceived and designed the experiments; Analyzed and interpreted the data; Contributed reagents, materials, analysis tools or data; Wrote the paper.

Zulfadlillah: Performed the experiments; Analyzed and interpreted the data; Contributed reagents, materials, analysis tools or data.

Muhammad Habiburrahman: Contributed reagents, materials, analysis tools or data; Wrote the paper.

### Funding statement

This work was supported by Hibah PUTI Q2 Universitas Indonesia 2020 Contract No. NKB1744/UN2.RST/HKP.05.00/2020.

### Data availability statement

Data will be made available on request.

### Declaration of interests statement

The authors declare no conflict of interest.

### Additional information

No additional information is available for this paper.

## Appendix A Supplementary data

Supplementary Material - Questionnaire.docx Supplementary Material - Questionnaire.docx
