# Supplementary material for: Relationship between ISO 9001:2015 and operational and business performance of manufacturing industries in a developing country (Indonesia)
Source: Heliyon. 2021 Jan 15;7(1):e05537. doi: 10.1016/j.heliyon.2020.e05537 (PMC7814109; doi:10.1016/j.heliyon.2020.e05537)
Supplement: Supplementary Material - Questionnaire.docx [file mmc1.docx]

Supplementary File

Questionnaire

Independent variable questionnaire design.

| Independent  Variable | No. | Question | Scale |
| --- | --- | --- | --- |
| Organizational leadership | 1 | Top management plans organizational goals and communicates them actively. | Likert 1 - 6 |
|  | 2 | Top management provides resources on managing and improving quality. | Likert 1 - 6 |
|  | 3 | Top management views that quality as the most important thing compared to production quantity. | Likert 1 - 6 |
|  | 4 | Top management determines quality as their responsibility. | Likert 1 - 6 |
|  | 5 | Top management regularly interacts with Quality Department and other department. | Likert 1 - 6 |
|  | 6 | Top management evaluate the achievement of quality performance. | Likert 1 - 6 |
|  | 7 | Top management anticipate the change and make plan to accommodate those change. | Likert 1 - 6 |
| Customer requirement | 1 | Customer requirements/Quality Standard of product have been identified and defined. | Likert 1 - 6 |
|  | 2 | Customer-oriented strategies are prepared and reviewed in an effort of continuous improvement. | Likert 1 - 6 |
|  | 3 | Design, development, and delivery of product fit to defined customer requirements. | Likert 1 - 6 |
|  | 4 | Customer satisfaction evaluation is conducted regularly. | Likert 1 - 6 |
|  | 5 | Customer complaints are documented, reviewed and followed up to improve quality. | Likert 1 - 6 |
| Defect prevention | 1 | Company designs efficient product and process. | Likert 1 - 6 |
|  | 2 | Quality audit is carried out to determine the conformity of products to specifications. | Likert 1 - 6 |
|  | 3 | Reducing defect problems through improved production, storage, packaging and shipping processes. | Likert 1 - 6 |
|  | 4 | Reducing defect problems through improved management of equipment related to the production process. | Likert 1 - 6 |
|  | 5 | Product and procedure control is carried out at all stages of production process. | Likert 1 - 6 |
| Continuous Improvement | 1 | The company determines what to focus on improving the quality management system. | Likert 1 - 6 |
|  | 2 | The company prepares an effective plan in an effort to improve quality. | Likert 1 - 6 |
|  | 3 | The company develops an organizational structure that support the continuous improvement of the quality system. | Likert 1 - 6 |
|  | 4 | Control and improvement of processes, procedures and products are carried out continuously. | Likert 1 - 6 |
|  | 5 | Apart of product quality, the quality of employees' work is continuously improved. | Likert 1 - 6 |
| Supplier Quality Management | 1 | In choosing supplier, the company considers product quality more important than price. | Likert 1 - 6 |
|  | 2 | The company certifies supplier and conduct regular audit to maintain quality standard. | Likert 1 - 6 |
|  | 3 | The company establishes a team that regularly visit to supplier to evaluate supplier product. | Likert 1 - 6 |
|  | 4 | The company has detailed data regarding supplier performance. | Likert 1 - 6 |
|  | *5* | Suppliers regularly receive feedback from the company in an effort to manage quality standard. | Likert 1 - 6 |

Dependent variable questionnaire design.

| Dependent variable | Question | Scale |
| --- | --- | --- |
| Business performance | Achievement of sales growth compared to the target. | Likert 1 - 6 |
|  | Achievement of market share growth compared to the target. | Likert 1 - 6 |
|  | Achievement of profit compared to the target. | Likert 1 - 6 |
| Operational performance | Achievement of productivity compared to the target. | Likert 1 - 6 |
|  | Achievement of quality product compared to the target. | Likert 1 - 6 |
|  | Achievement of customer satisfaction compared to the target. | Likert 1 - 6 |
